# Bungowannah Pestivirus Chimeras as Novel Double Marker Vaccine Strategy against Bovine Viral Diarrhea Virus

**DOI:** 10.3390/vaccines10010088

**Published:** 2022-01-07

**Authors:** Susanne Koethe, Patricia König, Kerstin Wernike, Jana Schulz, Ilona Reimann, Martin Beer

**Affiliations:** 1 Institute of Diagnostic Virology, Federal Research Institute for Animal Health, Friedrich-Loeffler-Institut, 17493 Greifswald-Insel Riems, Germany; Susanne.Koethe@fli.de (S.K.); Patricia.Koenig@fli.de (P.K.); Kerstin.Wernike@fli.de (K.W.); ilona.reimann@t-online.de (I.R.); 2 Institute of Epidemiology, Federal Research Institute for Animal Health, Friedrich-Loeffler-Institut, 17493 Greifswald-Insel Riems, Germany; Jana.Schulz@fli.de

**Keywords:** BVDV, pestivirus, DIVA vaccine, BuPV, chimera, N^pro^, E^rns^, modified live vaccine, inactivated vaccine

## Abstract

Marker or DIVA (differentiation of infected from vaccinated animals) vaccines are beneficial tools for the eradication of animal diseases in regions with a high prevalence of the designated disease. Bovine viral diarrhea virus (BVDV)-1 (syn. *Pestivirus A*) is a flavivirus that infects predominantly cattle resulting in major economic losses. An increasing number of countries have implemented BVDV eradication programs that focus on the detection and removal of persistently infected cattle. No efficient marker or DIVA vaccine is yet commercially available to drive the eradication success, to prevent fetal infection and to allow serological monitoring of the BVDV status in vaccinated farms. Bungowannah virus (BuPV, species *Pestivirus F*), a related member of the genus *Pestivirus* with a restricted prevalence to a single pig farm complex in Australia, was chosen as the genetic backbone for a marker vaccine candidate. The glycoproteins E1 and E2 of BuPV were substituted by the heterologous E1 and E2, which are major immunogens, of the BVDV-1 strain CP7. In addition, the candidate vaccine was further attenuated by the introduction of a deletion within the N^pro^ protein coding sequence, a major type I interferon inhibitor. Immunization of cattle with the chimeric vaccine virus BuPV_ΔN^pro^_E1E2 CP7 (modified live or inactivated) followed by a subsequent experimental challenge infection confirmed the safety of the prototype strain and provided a high level of clinical protection against BVDV-1. The serological discrimination of vaccinated cattle could be enabled by the combined detection of BVDV-1 E2- in the absence of both BVDV NS3- and BVDV E^rns^-specific antibodies. The study demonstrates for the first time the generation and application of an efficient BVDV-1 modified double marker vaccine candidate that is based on the genetic background of BuPV accompanied by commercially available serological marker ELISA systems.

## 1. Introduction

The successful development and application of a chimeric marker vaccine in combination with a DIVA (differentiation of infected from vaccinated animals) test strategy has been approved for many economically important animal pathogens such as bovine herpes virus type 1 (BoHV-1), suid herpesvirus type 1 (SuHV-1, syn. Aujeszky’s disease virus [ADV] or pseudorabies virus [PRV]) and *Pestivirus C* (syn. classical swine fever virus [CSFV]) [1,2,3]. The serological DIVA test strategy encompasses the implementation of differentiating serology tests (e.g., ELISAs) that clearly discriminate between antibodies acquired by field infection and vaccination. It has been pointed out that modern DIVA capable vaccines will improve disease eradication in comparison to standard vaccines, for example, by administration as an emergency vaccine in a pathogen-free country [4].

The genus *Pestivirus* of the *Flaviviridae* family encompasses a number of pathogen species of great economic importance [5] including CSFV and bovine viral diarrhea virus types 1 and 2 (BVDV-1, BVDV-2). New members of the genus *Pestivirus* have been found in recent years within a broad range of mammalian hosts—from livestock, rodents to bats and harbor porpoise [6,7,8,9,10,11].

BVDV is the causative agent of one of the most important diseases of cattle stock worldwide. It causes significant economic losses in the beef and dairy industries due to its impact on herd productivity and reproduction [12]. Two species of BVDV are described: BVDV-1 (*Pestivirus A*) and BVDV-2 (*Pestivirus B*) which are further subdivided in at least 21 subtypes within BVDV-1, and 4 within BVDV-2 [13]. According to their ability to induce apoptosis in infected cells, both species occur as noncytopathic (ncp) and cytopathic (cp) biotypes [14]. Clinical signs of an acute infection by any species can encompass respiratory, enteric and reproductive disorders. Disease manifestation may range from subclinical or mild signs to fatal disorders, characterized by for example, hemorrhagic syndromes (HS), or the late onset form mucosal disease (MD) that develops in persistently infected (PI) animals [15]. The primary reservoirs for BVDV in cattle worldwide are in utero infected, immunotolerant PI carriers that shed virus lifelong in high amounts [16,17]. Those PI calves can emerge after infection of pregnant animals during the first trimester of gestation with ncp BVDV [18].

BVDV control and eradication programs have been implemented particularly in Europe but also in further countries worldwide in the last decades [19,20,21]. The three pillars of the systematic BVDV control programs comprise: (i) the identification and elimination of PI cattle; (ii) increased surveillance of cattle herds by serological diagnostics to monitor the progress of intervention and to detect new infections; and (iii) measures to prevent the infection of pestivirus naïve animals and (re-) introduction of the virus into BVD-free herds [22]. Vaccination would assist as sustainable and valuable additional biosafety measure [22,23]; however, no serological marker vaccines are commercially available to date.

The members of the atypical pestivirus species *Pestivirus F*, the “Bungowannah virus” (BuPV), were first isolated in 2003 on a farm in New South Wales, Australia, that faced an outbreak of sudden deaths in piglets [6]. BuPV was shown to be associated with an incidence of stillbirths in piglets and was identified as the causative agent of the porcine myocarditis syndrome [24]. So far, no evidence for transmission into the environment or other regions or countries was found [25,26,27]. Sequence data collected from samples between 2010 and 2014 revealed a high genetic stability of the RNA genome [28]. Structural analyses demonstrated the absence of cross-reactivity for pan-pesti reactive monoclonal antibodies, suggesting the existence of major antigenic differences between BuPV and BVDV [29]. However, BuPV and BVDV envelope proteins are compatible, which has been demonstrated by complementation studies [30].

The overall genomic organization of all pestiviruses is similar. The single stranded RNA genome of positive polarity encodes for four structural proteins—the capsid C and the envelope proteins E^rns^, E1 and E2—and at least for eight nonstructural proteins (N^pro^, p7, NS2, NS3, NS4A, NS4B, NS5A and NS5B) [5]. Immunodominant proteins are the nonstructural protein NS3 and the glycoproteins E^rns^ and E2, as significant and detectable antibody titers against these proteins are produced in infected animals [31,32]. Neutralizing antibodies are mainly directed against E2, which mediates virus entry. Furthermore, E1 and E2 form heterodimers [33] and determine the host species tropism [34]. The immunoregulatory proteins N^pro^ and E^rns^ are unique to pestiviruses and mediate the evasion from the host’s interferon (IFN) response and the manipulation of the host’s self-non-self-discrimination for a successful establishment of persistent fetal infection and immunotolerance [35,36,37,38,39]. N^pro^ is an autoprotease and was shown to be nonessential for most of the pestiviruses [40]. In contrast, the glycoprotein E^rns^ is an essential structural component of the virus particle [41] and the mutation of its RNase activity leads to virus attenuation [42]. The introduction of mutations affecting E^rns^ RNase and N^pro^ protease, respectively, prevented persistent fetal infection [39].

Due to the high similarity in the genome organization of pestiviruses, individual genes are often interchangeable between different *Pestivirus* species. Several approaches have already shown the heterologous compatibility of pestivirus proteins resulting in the feasibility to generate pestiviral chimeras and their application for different purposes, for example, characterization of new viruses or viral proteins [43], development of serological assays for seroprevalence studies [44] and the development of marker vaccines [3,45,46]. As an example, the Suvaxyn CSF Marker^®^ vaccine (Zoetis Belgium SA, Louvain-la-Neuveb, Belgium) that contains live BVDV, which has been modified to replace the E2 gene of BVDV with the corresponding gene of CSFV, was market authorized in 2015. Numerous vaccination-challenge trials confirmed the efficacy and the safety of this chimeric pestivirus vaccine against CSFV genotypes 1 and 2 [47].

Here, the construction of a BVDV-1 double marker vaccine that is based on the genetic background of the atypical BuPV expressing the heterologous E1 and E2 glycoproteins of BVDV-1 is described. The double marker principle, that is defined by the presence of BVDV-1 E2-specific antibodies and the concurrent absence of BVDV-1 E^rns^- and NS3-specific antibodies upon immunization, and the protective capacity of the vaccination were investigated in a BVDV-1 vaccination-challenge trial in cattle.

## 2. Materials and Methods

### 2.1. Cells and Viruses

All cell lines used in the presented study were provided by the Collection of Cell Lines in Veterinary Medicine, CCLV, Friedrich-Loeffler-Institut (FLI), Greifswald-Insel Riems. The diploid bovine esophageal cell line KOP-R (RIE244) was used for electroporation of infectious RNA, virus rescue, growth kinetics and neutralization assays. Madin–Darby bovine kidney (MDBK) cells (RIE261) were used for virus titration and the IFN-incompetent MDBK41 cells (RIE728) were used for virus propagation of BuPV_ΔN^pro^_E1E2 CP7.

The cytopathic BVDV-1b strain CP7 was isolated from a case of fatal mucosal disease [48]. SE5508, a ncp BVDV-1b strain, was isolated from a clinically healthy immunotolerant calf and used for the heterologous challenge infection and the BVDV neutralization assay.

### 2.2. Generation of BuPV_ΔN^pro^_E1E2 CP7 Virus and Virus Recovery

The generation of the infectious full-length cDNA clone of BuPV (pA/BV) (GenBank accession number: NC_023176) has been described in detail in the publication of Dalmann et al. [28]. The construct BuPV_ΔN^pro^_E1E2 CP7 was generated by the deletion of most of the coding genomic region of N^pro^ (nucleotides [nt] 428–922, amino acids [aa] 13–165) and by substitution of the glycoproteins E1 and E2 encoding genomic region with E1 and E2 of BVDV-1b strain CP7 (GenBank accession number: BVU63479). The deletion and substitution were achieved. For this purpose, the genomic region of CP7_E1E2 was amplified and extended by overlapping sequences of BuPV using Phusion High-Fidelity Polymerase (New England Biolabs GmbH, Frankfurt am Main, Germany) according to the manufacturer’s instruction. These amplification products served as megaprimers and were used to delete and substitute BuPV_E1E2 in the BuPV backbone by Fusion PCR methods [49].

Plasmids were amplified in *Escherichia coli* DH10B™ cells (Thermo Fisher Scientific, Darmstadt, Germany). Using the Qiagen Plasmid Midi Kit (Qiagen, Hilden, Germany), the plasmid DNA was purified. The correct sequence was confirmed by Sanger termination cycle sequencing using a Big Dye Terminator Mix (Applied Biosystems, Thermo Fisher Scientific). Primer sequences and protocols are available on request. Sequencing products were purified with a NucleoSEQ Spin kit (Macherey-Nagel, Düren, Germany) and sequenced on an ABI 3500 Genetic Analyzer (Applied Biosystems). To recover virus, the cDNA construct was linearized by *SmaI* digest and in vitro transcribed with the T7 RiboMax Large Scale RNA Production System (Promega, Walldorf, Germany). The in vitro transcribed RNA was electroporated into SK6 cells [50]. The supernatant of electroporated cells was harvested 72 h later and was tested for the presence of infectious virus. Recovered virus was serially passaged on MDBK41 cells.

### 2.3. Growth Kinetics

To determine the growth kinetics of the vaccine candidate BuPV_ΔN^pro^_E1E2 CP7 in comparison to wild type BuPV in vitro, KOP-R cells were inoculated at a multiplicity of infection (m.o.i.) of 1. Supernatants were collected at 0, 24, 48 and 72 h post-infection (p.i.) and titrated on MDBK cells. The titers were calculated and expressed as 50% tissue culture infectious dose per ml (TCID_50_/mL). Growth kinetics were repeated twice.

### 2.4. Ethics, Animals and Experimental Design

The animal study was conducted in compliance with governmental animal welfare guidelines and regulations and was approved by the State Office of Agriculture, Food safety and Fishery in Mecklenburg-Western Pomerania under the registration number 7221.3-1-034/15.

Fifteen conventionally reared female Holstein–Friesian calves obtained from local farms were included in the vaccination and challenge study after being tested negative for the presence of BVDV- and BoHV-1-specific antibodies. The animals were allocated into three different groups for the trial with a group size of five animals each. The animals were housed in the biosafety level-2 facility of the FLI. Their age ranged from four to seven months with an equal distribution in each group. Animals were vaccinated once or twice at an interval of four weeks. Four weeks after the final immunization, both groups as well as the non-vaccinated control group were intranasally (i.n.) infected with the BVDV-1 challenge strain SE5508. For BVDV-1 vaccination, the animals were either vaccinated intramuscularly (i.m) once with live virus BuPV_ΔN^pro^_E1E2 CP7 adjusted to 5 × 10^5^ TCID_50_ or in two vaccination cycles with an inactivated vaccine at day 0 (first vaccination, V1) and at day 28 (second vaccination, V2) with 1 mL containing an equivalent of 5 × 10^5^ TCID_50_ viral particles adjuvanted with 20% polygen. Back titrations verified a dose of 1 × 10^6^ TCID_50_ of the live vaccine and 2.8 × 10^5^ TCID_50_ for each vaccination with the binary ethylenimine (BEI)-inactivated vaccine (titration before inactivation). For the BEI-inactivation, 210 mg 2-bromoethylammoniumbromide (BEA [Merck, Darmstadt, Germany]) was solved in 9 mL aqua dest. in the presence of 1 mL 2N NaOH and heated at 37 °C for 1 h to allow cyclisation of BEA to BEI. The final concentration of BEI was adjusted to 4 mM. Inactivation of the virus preparation was accomplished through a continuous inverting at 37 °C for 24 h. Sodium thiosulphate was finally added in a concentration of 4% to inactivate the excess of BEI. Complete inactivation was verified by inoculation on KOP-R cells. Two consecutive blind passages of supernatants were performed 3 days after inoculation.

For the i.n. BVDV-1 challenge infection at day 56, 2 mL (1 mL per nostril) with 2 × 10^7^ TCID_50_ of the SE5508 strain were administered with a diffusor. Data of the control group have already been published in Koethe et al., 2020 [46].

### 2.5. Clinical Evaluation

Rectal body temperatures were measured daily and clinical examinations were carried out daily during the course of the trial. The calves were examined for adverse reactions immediately after vaccination and challenge infection. Signs of clinical disease, focusing on respiratory and digestive disorders, and the general health status (depression, feed intake and behavior) were controlled.

### 2.6. Nasal Swabs and EDTA-Blood Samples

Over the period of 10 days after the respective first vaccination and for 14 days after the challenge infection, nasal swabs and EDTA-blood samples were collected daily. To monitor the serological response, serum samples were taken weekly. The samples were subjected to hematological, virological and serological analyses.

### 2.7. Hematological Investigations

Blood samples were taken by jugular venipuncture and were collected in potassium EDTA-coated sterile blood collection tubes (Monovette) (Sarstedt, Nürnbrecht, Germany). To determine the differential blood cell counts, an Abbott CellDyn 3700 analyzer (Abbott, Wiesbaden, Germany) was used.

### 2.8. Virus Isolation

The isolation of virus using cell culture was essentially performed as described by Zemke et al., 2010 [51]. Virus isolation was conducted on nasal swabs and on concentrated and purified Peripheral Blood Mononuclear Cells (PBMC) after ammonia lysis of red blood cells. Monolayers of KOP-R cells were inoculated in four replicates per animal and specimen. Virus replication was confirmed by indirect immunofluorescence staining of the viral NS3 protein using mab mix WB103/105 (c.c.pro, Oberdorla, Germany) and an Alexa488 goat anti-mouse IgG conjugate (Thermo Fisher Scientific). Evaluation was carried out using the fluorescence microscope Nikon Eclipse Ti (Nikon, Düsseldorf, Germany). One blind passage of the supernatants was performed after 3 to 4 days of inoculation.

### 2.9. Serology

Sterile blood collection tubes (Monovette) with clot activator were used for weekly intravenous serum sampling. After centrifugation at 3,000 rpm, serum aliquots were stored at −20 °C. All sera were incubated at 56 °C for 30 min prior to serological testing.

To perform serological investigations, the following ELISAs were used: ID Screen^®^BVD p80 antibody competition ELISA (IDVet, Grabels, France), Monoscreen Ab ELISA BVDV (E0) (Bio-X Diagnostics S.A., Rochefort, Belgium) and IDEXX BVDV Total Ab ELISA (IDEXX, Liebefeld-Bern, Switzerland). To process the samples, the manufacturer’s instructions were followed. For the ID Screen^®^BVD p80 antibody competition ELISA, the relative blocking values (%S/N) were calculated and samples with %S/N ≤ 40% were considered as positive, between 40% and 50% as suspicious and ≥ 50% as negative according to the recommendation of the manufacturer. For the Monoscreen Ab ELISA BVDV (E0), all inhibition E^rns^ values (%) were calculated and samples with inhibition of E^rns^ values (%) ≥ 50% were considered as positive as recommended by the manufacturer. For the BVDV Total Ab ELISA, S/P values < 0.20 are considered as negative, between 0.2 and 0.3 as suspicious (intermediate) and ≥0.3 as positive. In our study, all suspicious (intermediate) test results were classified as positive.

Sera from all animals were tested in a standard neutralization assay (NA) against homo- and heterologous BVDV-1 (CP7 and SE5508) and BuPV. The NA was conducted as described by Zemke et al., 2010 [51]. Titers were expressed as reciprocal of the highest dilution that caused 50% neutralization per ml (ND_50_/mL).

### 2.10. Statistics

To compare whether the means of the cumulated scoring values are significantly different between the three groups (unvaccinated control group, BuPV_ΔN^pro^_E1E2 CP7 live 1× and BuPV_ΔN^pro^_E1E2 CP7 inactivated 2×) a one-way analysis of variance (one-way ANOVA) was performed based on the results for virus isolation from nasal swabs as well as for virus isolation from leukocytes. The cumulated scoring values were normally distributed assessed by the Shapiro-Wilk test. Homogeneity of variances was determined using Bartlett’s test and no outliers were observed. Thus, the assumptions for the one-way ANOVA were fulfilled. Dunnett’s test was used as post-hoc test to determine which group means were significantly different from the unvaccinated control group. For all statistical tests, a significance level of 5% was assumed. Statistical analysis was performed in R (version 4.0.3) [52].

## 3. Results

### 3.1. Construction and Characterization of BuPV_ΔN^pro^_E1E2 CP7

**Figure 1:**
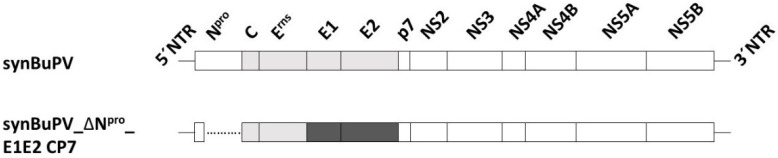
Schematic overview of the synthetic recombinant marker vaccine virus BuPV_ΔN^pro^_E1E2 CP7 (lower panel) based on the BuPV backbone (upper panel). The structural proteins are shown in light grey boxes and nonstructural proteins in white boxes. The inserted E1 and E2 proteins of BVDV-1 CP7 are depicted in dark grey boxes (lower panel).

BuPV_ΔN^pro^_E1E2 CP7 was constructed synthetically on the genetic background of BuPV. The E1 and E2 glycoprotein sequences of BuPV were substituted with the E1 and E2 encoding genomic region of BVDV-1b CP7 strain to obtain a BVDV-1 vaccine candidate. In line with a comprehensive vaccine strategy and the published BVDV-1 and BVDV-2 chimeric marker candidate vaccines [46], the viral IFN antagonist N^pro^ was deleted with retaining only the first 12 aa to ensure a correct processing of the polyprotein in combination with a stable attenuation of the vaccine candidate. To discriminate vaccinated from BVDV-field infected animals, the genetic sequences of the glycoproteins E^rns^ and the nonstructural protein NS3 of BuPV were retained (Figure 1).

After RNA transfection, the chimeric virus was rescued on KOP-R cells and passaged on MDBK41 cells. The co-expression of BuPV E^rns^ and BVDV-1 E2 were confirmed in infected cells by immunofluorescence staining using appropriate specific antibodies (data not shown).

Growth kinetics of BuPV_ΔN^pro^_E1E2 CP7 were performed with an m.o.i. of 1. Delayed virus growth with reduced final titers for BuPV_ΔN^pro^_E1E2 CP7 on bovine cells in comparison to the parental BuPV was detected (Figure S1). Furthermore, the genomic and sequence integrity of the rescued virus and the genetic stability of the viral sequence after 15 serial passages in cell culture could be confirmed by Sanger sequencing and high-throughput sequencing approaches (data on request).

### 3.2. Vaccination of Cattle by Administration of BuPV_ΔN^pro^_E1E2 CP7

The potential of BuPV_ΔN^pro^_E1E2 CP7 as a new BVDV marker vaccine candidate was analyzed by testing two different immunization strategies. The virus was administered i.m. once as a live vaccine (one shot: 1 × 10^6^ TCID_50_) or twice as a BEI-inactivated, polygen adjuvated virus preparation (two shots, each corresponding to 2.8 × 10^5^ TCID_50_). No virus could be re-isolated from nasal swabs or purified leukocytes after vaccination in both groups. The rectal body temperatures and the leukocyte counts remained stable and no adverse reactions or clinical signs like nasal discharge or diarrhea were observed in both groups. The quantity of BVDV-E2 specific antibodies produced upon vaccination was specified in a serum neutralization assay against the E1/E2-homologous CP7, the heterologous challenge virus BVDV-1 SE5508 strain and the parental BuPV.

**Figure 2:**
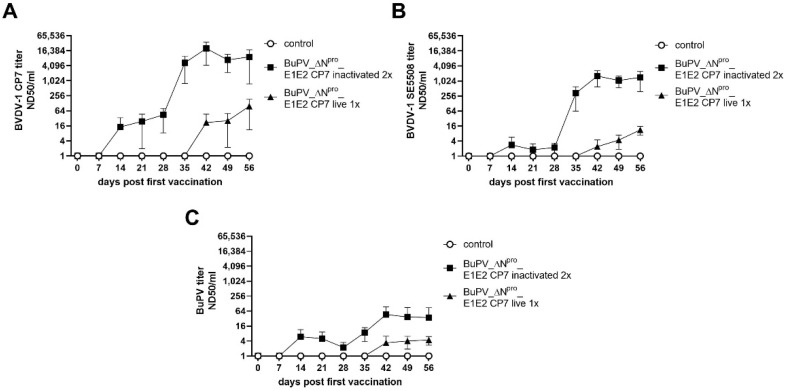
Development of neutralizing antibody titers after vaccination with BuPV_ΔN^pro^_E1E2 CP7. Mean neutralizing antibody titers against the homologous strain BVDV-1 CP7 (**A**), the heterologous (challenge) strain BVDV-1 SE5508 (**B**), and BuPV (**C**) were determined after one shot vaccination with live (live 1x) (28 days post first vaccination of the one-shot group) or two shots vaccination with inactivated (inactivated 2x) (0 and 28 days post first vaccination) BuPV_ΔN^pro^_E1E2 CP7.

**Figure 3:**
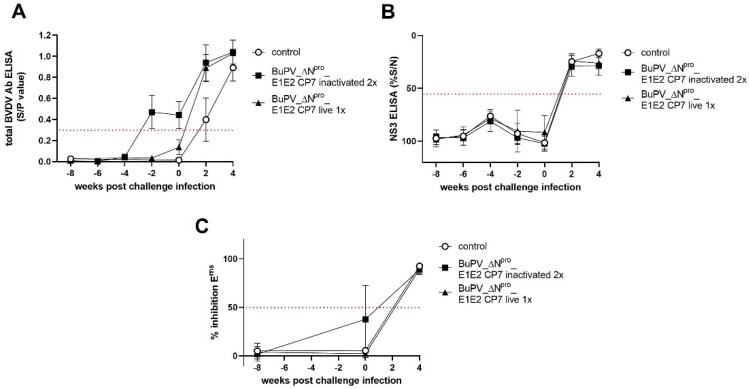
Kinetics of BVDV E2−-, NS3−- and E^rns^−-specific antibodies after vaccination and challenge infection. ELISA testing was performed on BuPV_ΔnproΔN^pro^_E1E2 CP7−-vaccinated and BVDV-1 challenged cattle. The animals were either vaccinated with one (1x×) shot of live or two (2x×) shots of inactivated candidate vaccine. A total BVDV Ab ELISA was performed (**A**) to monitor the serological response after vaccination and challenge infection. The presence of NS3−- and E^rns^−-specific antibodies were determined by a competitive NS3 (p80) antibody ELISA (**B**) and a competitive E0 (E^rns^) antibody ELISA (**C**). Relative blocking values (% S/N (B), % inhibition E^rns^ (**C**)) are indicated as mean group values. Error bars represent standard deviations. The cut-off values of the tests are marked through dotted lines.

Initial vaccination with the inactivated BuPV_ΔN^pro^_E1E2 CP7 resulted in the detection of BVDV-1b-specific neutralizing antibodies with titers of up to 1:44 against the homologous CP7 strain. The second vaccination clearly boosted the antibody levels leading to titers of up to 1:20,000. In comparison, the neutralizing titers against BuPV reached levels of only up to 1:46 after the second vaccination. Vaccination with the modified live vaccine candidate resulted in BVDV-1b neutralizing antibody titers of up to 1:98, and 1:4 against BuPV. No BVDV NS3-specific antibodies were detected by ELISA in the inactivated or in the live BuPV_ΔN^pro^_E1E2 CP7-vaccinated animals fulfilling the requirements for a marker vaccine. All animals of the control group remained negative in all applied tests (Figure 2A–C and Figure 3B).

### 3.3. BVDV-1 Challenge Infection of BuPV_ΔN^pro^_E1E2 CP7-Vaccinated Cattle

**Figure 4:**
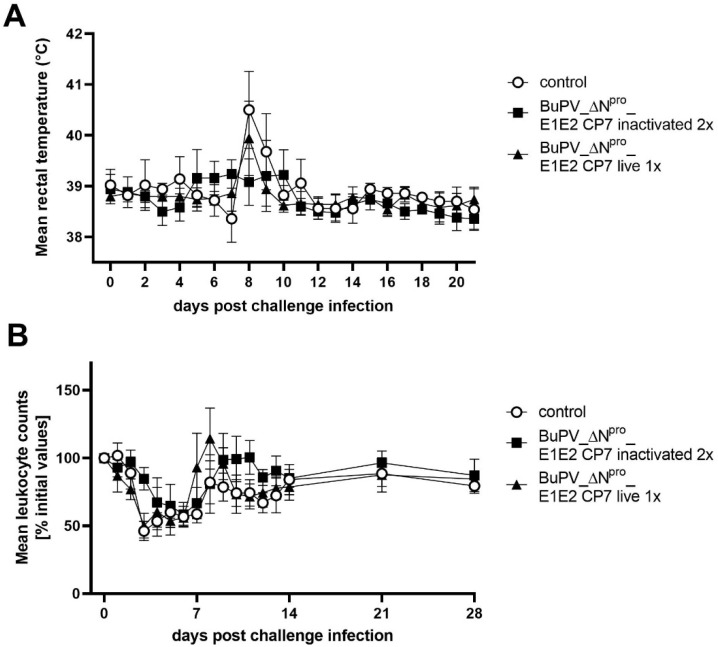
Mean rectal body temperatures and kinetics of blood leukocyte counts after BVDV-1 challenge infection of BuPV_ΔN^pro^_E1E2 CP7-vaccinated cattle. The animals were vaccinated with one shot of live (live 1x) or two shots of inactivated (inactivated 2x) BuPV_ΔN^pro^_E1E2 CP7. (**A**) Mean group values of the rectal body temperature after the challenge infection are depicted. Temperatures lower than 39.5 °C are considered as physiological temperature, higher than 40 °C as fever. (**B**) Course of mean blood leukocyte counts after the challenge infection is presented. Mean values of the different groups are shown in percentage of the initial values (number of leukocytes measured at the day of challenge infection). The initial values (measured value at day of challenge) were set to 100%. Error bars represent standard deviations.

At day 56 of the trial, 28 days after the last vaccination, all animals were infected i.n. with the heterologous BVDV-1 strain SE5508. All unvaccinated animals of the control group developed typical and clear clinical signs of infection. The rectal body temperatures showed a monophasic rise at day 7 post challenge infection (p.chall.) with a maximum mean group value of 40.5 °C (Figure 4A). Animals of the live vaccinated group showed on day 7 p.chall. a moderate elevation of the rectal body temperatures slightly exceeding the physiological temperature range (peak at 39.9 °C). In contrast, inactivated BuPV_ΔN^pro^_E1E2 CP7-vaccinated animals showed only a mild elevation of the body temperatures above 39 °C over a period of six days starting at day 4 p.chall. with mean body temperatures of 39.08–39.24 °C compared to a pre-challenge mean of 38.75 °C. Besides the elevated rectal body temperature, only minor additional clinical effects were in general observed after the SE5508 challenge infection. The control animals showed a slightly reduced feed intake and a marginally reduced general condition for one day (7 or 8 d p.chall.). Diarrhea was not observed and respiratory distress did not exceed the pre-challenge observations. The vaccinated animals of both groups remained completely asymptomatic.

In addition to the symptoms observed during clinical examination, all control animals showed a severe leukopenia. The triphasic decrease (3, 6 and 12 d p.chall.) in leukocyte counts showed a maximum mean level in reduction of 54% on day 3 p.chall. (Figure 4B). The live BuPV_ΔN^pro^_E1E2 CP7 vaccinated animals also showed a leukopenia with a triphasic decrease (3, 5 and 11 d p.chall.) and revealed a comparable mean level in leukocyte counts reduction of 52%. The leukocyte counts normalized in the control and live vaccinated group on day 8 and day 7 p.chall, respectively. In contrast to the unvaccinated control group, a short term (one day) rebound in leukocyte counts was detected for the live vaccinated animals as early as by day 8 p.chall. Animals of the inactivated BuPV_ΔN^pro^_E1E2 CP7 vaccinated group showed a biphasic decrease in leukocyte counts (6 and 12 d p.chall.). The maximum mean level in leukocyte counts reduction was 41% on day 6 p.chall. Moreover, a rebound in leukocyte counts was shown for the inactivated BuPV_ΔN^pro^_E1E2 CP7 vaccinated group on day 9 p.chall. with restoration of the pre-challenge values (Figure 4).

Nasal shedding of infectious virus and a transient viremia may occur during an acute infection with BVDV-1. Therefore, virus isolation from nasal swabs and from blood was conducted daily starting on day 1 p.chall. until day 14 p.chall. In the control animals, long and pronounced nasal shedding of challenge virus and viremia were found. Virus was secreted from the nose of every animal of this group on five to eight consecutive days, whereas virus was isolated from nasal swabs on two to five coherent days in the live vaccinated and only on a maximum of two coherent days in the group that received the inactivated vaccine. In addition, virus was isolated over a period of 11 days from the leukocytes in the control group. In contrast, only on two or three consecutive days virus was isolated from the leukocytes of a few animals of the vaccinated groups, respectively (Table 1). Clearly, the two shots vaccination with the inactivated BuPV_ΔN^pro^_E1E2 CP7 protected one out of five animals completely from nasal shedding and two out of five from viremia. When shedding and viremia occurred, both events were only detectable on a single or maximum two days. In summary, both the nasal shedding of infectious virus and the viremia were significantly reduced in both vaccinated groups in comparison to the unvaccinated control group (*p* < 0.001).

### 3.4. Marker Serology

The challenge infection boosted the presence of BVDV E2-specific antibodies, as determined by a commercial E2-based ELISA (Figure 3A). In the control group, BVDV E2-specific antibodies were first detected two weeks p.chall. In line, the presence of NS3-specific antibodies in the sera of unvaccinated control and vaccinated animals was first observed at two weeks p.chall. confirming the absence of BVDV NS3–specific antibodies after vaccination and the applicability of the ELISA to discriminate BVDV NS3 and BuPV NS3 antibodies. Furthermore, the commercially available competitive BVDV E0 (E^rns^) antibody ELISA could also discriminate between vaccinated and challenge-infected animals (Figure 3A–C). However, certain levels of cross reactivity against BuPV E^rns^ were observed upon vaccination.

## 4. Discussion

Vaccines assisted for decades in control and eradication programs for economically important animal pathogens such as BoHV-1 or SuHV-1. For BVDV, eradication programs have been implemented in several countries worldwide and the availability of a DIVA-compatible vaccine could contribute significantly in regions of high virus prevalence to drive back field virus prevalence [20]. Furthermore, a marker vaccine could be applied as an emergency vaccine in BVDV-free countries, where control and eradication are major objectives when the disease is introduced and where serological methods are essential to demonstrate freedom of disease once the outbreak is under control. However, none of the currently commercially available BVDV vaccines allows the establishment of a serological DIVA strategy and the differentiation of vaccinated from field-infected animals by serological methods. They only serve as genetic DIVAs as the vaccine virus can be differentiated using RT-PCR and/or sequencing methods [53].

The overall aim of this study was to develop a marker vaccine prototype for BVDV-1 using a novel approach based on a synthetic backbone strategy of a related pestivirus with chimeric protein expression that has successfully been employed previously [46]. The synthetic backbone strategy allows the rapid exchange, deletion or insertion of sequences or genes. The modified novel approach encompassed the utilization of the atypical pestivirus BuPV as the genetic background because of its spatial restriction to a single pig farm complex in Australia [25], the presence of major antigenic difference between BuPV and BVDV-1 [29], and the compatibility of BuPV and BVDV envelope proteins [30]. To allow attenuation of the vaccine candidate, the gene of the IFN antagonist N^pro^ protein was partially deleted. To test the safety and efficacy of the novel potential marker vaccine candidate BuPV_ΔN^pro^_E1E2 CP7, calves were experimentally vaccinated and subsequently challenged with a respective heterologous BVDV-1 field strain. In accordance with the prospective marker principle, immunization of cattle with the vaccine candidate should induce seroconversion of BVDV-1 E2-specific antibodies while BVDV-specific NS3- and E^rns^-antibodies should only be detectable after BVDV-1 challenge infection. This feature makes the novel vaccine candidate to our knowledge the first “double marker vaccine” against BVDV.

After virus recovery, virus growth kinetics revealed a 100–1,000-fold less efficient growth of BuPV_ΔN^pro^_E1E2 CP7 in comparison to the wildtype BuPV. The observed in vitro attenuation of BuPV_ΔN^pro^_E1E2 CP7 on bovine cells is most likely due to a combination of two factors—the expression of a heterologous heterodimer of E1 and E2 and the absence of the immunomodulatory N^pro^ protein. A recent study characterizing a CSF mutant virus has already shown that the deletion of N^pro^ and the absence of the immunosuppressive function led to an attenuation in vivo [54]. Despite that, most of the N^pro^ encoding sequences were deleted in our construct in order to further increase the safety for pregnant dams, a concept already applied for the commercial BVDV vaccine Bovela^®^ (Boehringer Ingelheim Vetmedica GmbH, Ingelheim/Rhein, Germany) and the previously described marker vaccine candidates BVDV-1b_synCP7_ΔN^pro^_E^rns^ Bungo and BVDV-1b_synCP7_ΔN^pro^_E^rns^ Bungo_E1E2CS [46]. Regarding the factor of the heterologous heterodimer of E1 and E2 that might negatively influence the in vitro growth of BuPV_ΔN^pro^_E1E2 CP7, we selected the combined replacement of both proteins because of the high genetic diversity between BuPV and BVDV-1 and as the formation of E1-E2 heterodimers is essential for virus entry [55]. The overall amino acid homology of the polyprotein between BVDV-1 (strain CP7) and BuPV is 52.6%, whereas the amino acid sequence identities within the E1 of BVDV-1 and BuPV only amount to 44.9% and within the E2 to 41.2%. Previous complementation studies showed that only the homologous co-expression of BuPV-E1 and E2 in a BVDV backbone resulted in recovery of infectious virus [30]. The heterodimer E1E2 is stabilized through the formation of disulfide bonds [33]. The glycoprotein E1 of BVDV_CP7 and BuPV differ in the number of cysteine residues (BVDV_CP7 E1: 6 residues vs. BuPV E1: 3 residues). Therefore, a single substitution of either E1 or E2 in chimeras may hamper the heterodimer formation. This is in contrast to the commercially available Suvaxyn CSF Marker^®^ vaccine (CP7_E2alf) where only the genetic sequence of the E2 protein of BVDV-1 was substituted with CSFV E2alf (strain Alfort/187). The E2 of CSFV and BVDV-1 share 58.3% amino acid identities and the structure of BVDV E2 is similar to that of CSFV, which might explain the interchangeability [56]. Resolving and comparing the crystal structure of BuPV E2 with BVDV and CSFV would help to explain whether a functional interchangeability of the glycoproteins E1 and E2 is only a matter of structure.

The utilization of BuPV as the backbone for the marker vaccine may rise safety concerns as the vaccination of cattle might lead to spillover infection of the vaccine candidate BuPV_ΔN^pro^_E1E2 CP7 to other susceptible species. Previous studies have already determined the susceptibility and the clinical outcome of BuPV infection in various cloven-hoofed species and interspecies transmission from chronically infected pigs. Cattle and sheep appeared to be less susceptible to infection in comparison to pigs, and BuPV was failing to establish immunotolerance and persistence in the bovine fetus. The authors of the study argued that the transmission of BuPV is likely to be inefficient in ruminants and will probably not sustain in these species [57]. Moreover, no nasal shedding of the vaccine candidate occurred after immunization in the present study minimizing the potential for any spillover infections to contact cattle or pigs. The deletion of most of the encoding sequencing of the IFN antagonist N^pro^ in the BVDV-1 CP7 genetic background reduced virus titer and induced a strong IFN response in cell culture [46]. The abrogation of its immunosuppressive function through the deletion of N^pro^ in CSF mutant viruses led to an attenuation in vivo [54]. Therefore, the deletion of N^pro^ in the presented double marker vaccine is an important factor of safety. Nevertheless, safety aspects of the BuPV_ΔN^pro^_E1E2 CP7 vaccine candidate need to be further investigated by vaccination of pregnant cows and in utero infection of growing fetuses to test for any possible induction of persistence or abortion and further determination of the susceptibility of pigs.

The application of numerous inactivated or killed and modified-live vaccines (MLV) against BVD have been described before. MLVs are considered to be efficacious and induce a strong humoral and cellular immunity and provide a solid fetal protection as immunogenic proteins are amplified through viral replication. However, there are reports about adverse effects that can be attributed to the immunosuppressive effects of live vaccines and safety issues with vaccination in pregnancy [18,58]. The development of inactivated or killed vaccines (KV) were prompted because of the safety concern for MLV. Furthermore, the detection of live vaccine viruses in tissue samples of newborn calves whose mothers were immunized within the first trimester of gestation [19,53] and the interference with the BVD ear notch diagnostics underline the need for a further safe and efficient vaccine to support BVDV control programs. The application of KV induces immune responses that are limited basically to antibody production towards the structural proteins in the absence of a profound induction of CD8+ cytotoxic T cells (CTL) [59]. However, the control and clearance of BVDV infection are facilitated by both antibody and CTL responses preventing clinical disease and fetal infection [60]. The higher efficacy of MLVs in comparison to KV against BVD can be explained by (i) production of antibodies against the structural E2 and nonstructural protein NS3, and (ii) the induction of BVDV-specific CD8^+^ T cells. A recent study demonstrated the presence of novel potent IFN-γ inducing CD8^+^ T cell epitopes in the structural BVDV antigens (Ag) of E^rns^, E1 and E2 and in the non-structural Ags of N^pro^, NS2-3, NS4A-B and NS5A-B, and showed that these epitopes are highly conserved among more than 200 strains from BVDV-1 and -2 genotypes [61]. In the light of these observations, the usage of BuPV as a heterologous genetic backbone for the development of a live BVD vaccine may reduce the efficacy because only the properties of structural BVD E1 and E2 Ag as CD8^+^ T cell epitopes are retained. In contrast, a study has shown that the application of a single shot of Suvaxyn CSF Marker^®^ vaccine, the licensed CP7_E2alf MLV, resulted in a significant reduction of proinflammatory cytokine levels upon challenge, especially TNF-α and IL-6, and suggested an important role of cell-mediated immunity in both short- and long-term protection against CSFV [62]. Whether the application of live BuPV_ΔN^pro^_E1E2 CP7 stimulates the cell-mediated immunity and reduces proinflammatory cytokine levels upon BVDV challenge would be interesting to study.

The advantage of inactivated vaccines is the risk-free application at any age or stage of pregnancy. Furthermore, an inactivated vaccine is highly favored to be applied as an emergency vaccine in BVD-free countries as an accidental dissemination is impeded in contrast to live vaccines. On the downside, these vaccines are less efficient in comparison to MLV and at least one booster vaccination is required to achieve full protection. To balance the safety concerns and the efficacy, the chimeric vaccine candidate BuPV_ΔN^pro^_E1E2 CP7 was tested as inactivated preparation applied twice in addition to the application as a live vaccine with only one shot. In comparison to the single live vaccination, the inactivated booster vaccination even protected from moderate rectal body temperature elevation (39.94 vs. 39.24 °C) and delayed the leukopenia (3 vs. 6d p.i.) and their severity (48.8 vs. 58.6% mean leukocyte count). Nevertheless, both vaccination regimes, that is, the one shot live and the two shots of the inactivated chimeric vaccine BuPV_ΔN^pro^_E1E2 CP7, provided clinical protection against a virulent BVDV-1 challenge infection in the bovine host. Vaccination was efficient to confer a significant reduction of the nasal viral shedding and systemic replication shown by cell bound viremia in extent and duration. After double vaccination infectious challenge virus could not be recovered from blood samples of two out of five animals, however an actual sterile immunity was not induced as anti-NS3 and anti-E^rns^ specific antibodies were detectable in all vaccinated animals following challenge infection.

Twenty-eight days after the first vaccination, both groups developed neutralizing antibodies against the heterologous challenge strain. Most importantly, no antibodies specific for BVDV-1 NS3 or E^rns^ were found upon vaccination, facilitating the differentiation of immunized cattle from field-virus infected animals using a double marker principle which would be especially suitable under field conditions. Hence, the vaccination success could easily be monitored by using different commercially available and field-proven ELISA tests that can differentiate BuPV E^rns^ from BVDV E^rns^ and BuPV NS3 from BVDV NS3-specific antibodies, respectively, without interference of any cross-reactions. The challenge infection induced BVDV E^rns^- and BVDV NS3-specific antibodies in the controls and in the vaccinated groups, confirming the applicability of the double marker vaccine strategy to discriminate vaccinated from BVDV-1-infected animals. Furthermore, local and systemic field virus replication is significantly reduced which allows us to expect efficient suppression of field virus spread and prevention of fetal infection.

## 5. Conclusions

The application of the pestivirus BuPV as the genetic backbone for the design of a BVDV-1 double marker vaccine and the substitution of BuPV_E1E2 with BVDV-1_E1E2 resulted in the induction of a robust immune response that protected from clinical disease after BVDV-1 challenge infection. A clear serological discrimination of vaccinated from infected cattle was confirmed through the detection of BVDV-1 E2 in the absence of BVDV NS3 and BVDV E^rns^-specific antibodies. Independent of the applied vaccination regime (one shot live or two shots inactivated), BuPV_ΔN^pro^_E1E2 CP7 provided protection from fever, and the animals experienced a shorter period of leukopenia and a subsequent rebound of leukocyte numbers. Most importantly, viremia and nasal shedding were significantly reduced in extent and in duration in the vaccines compared to the control group. However, sterile immunity could not be achieved with this type of vaccine. A first orientation study about potencies and risks of the live vaccine virus was conducted to gain basic insights into characteristics and safety aspects. Overall, double immunization with the inactivated marker virus was more efficient to reduce viremia and virus shedding in comparison to the one-shot live vaccine and might be preferred in future applications. Furthermore, the application of the inactivated double marker vaccine is advantageous in countries with control programs like Germany because vaccinated animals remain seronegative in the standard commercial NS3-specific ELISA.

## Figures and Tables

**Table 1:** Virus shedding and isolation from nasal swabs and leukocytes after the BVDV-1 challenge infection. The cattle were vaccinated with one shot of live (live 1x) or two shots of inactivated (2x inactivated) BuPV_ΔN^pro^_E1E2 CP7. Highly BVDV-susceptible KOP-R cell cultures were inoculated with four replicates of fluids from nasal swabs (A) and purified leukocytes (B) from EDTA-blood samples (100 µL per replicate). The virus replication was verified by immunofluorescence staining three days p.i.. Results were scored according to the number of positive inoculations out of the four replicates (0 = no BVDV isolation, 4 = all inoculations BVDV positive) and visualized using light (1) or dark grey (4) colored fields. A first result was confirmed after one blind passage of the supernatants of the first cell culture inoculation.

(A) Virus Isolation from Nasal Swabs																
		Days post challenge infection														cumulated
Group	Animal no.	1	2	3	4	5	6	7	8	9	10	11	12	13	14	scoring values
Unvaccinated	401	0	1	3	1	4	4	4	2	0	0	0	0	0	0	19
control	403	4	3	0	1	2	3	2	1	0	0	0	0	0	0	16
	407	1	4	2	1	1	1	2	2	0	0	0	0	0	0	14
	413	1	4	4	1	3	4	3	0	0	0	0	0	0	0	20
	876	0	4	1	3	1	4	2	1	0	0	0	0	0	0	16
																**mean score 17**
BuPV_ΔN^pro^_E1E2 CP7	408	0	4	3	0	3	0	0	0	0	0	0	0	0	0	10
live 1x	421	0	4	0	0	1	1	0	0	0	0	0	0	0	0	6
	891	0	2	2	0	0	0	0	0	0	0	0	0	0	0	4
	896	0	4	2	2	1	3	0	0	0	0	0	0	0	0	12
	897	0	1	0	0	3	2	0	0	0	0	0	0	0	0	6
																**mean score 7.6**
BuPV_ΔN^pro^_E1E2 CP7	425	0	0	1	0	0	0	0	0	0	0	0	0	0	0	1
inactivated 2x	889	0	0	0	0	0	0	0	0	0	0	0	0	0	0	0
	890	0	1	0	0	0	0	0	0	0	0	0	0	0	0	1
	892	0	1	0	0	1	0	0	0	0	0	0	0	0	0	2
	899	2	1	0	0	0	0	0	0	0	0	0	0	0	0	3
																**mean score 1.4**
**(B) Virus Isolation from Leukocytes**																
		Days post challenge infection														cumulated
Group	Animal no.	1	2	3	4	5	6	7	8	9	10	11	12	13	14	scoring values
Unvaccinated	401	0	0	1	1	4	4	3	2	1	1	0	0	0	0	17
control	403	0	2	0	1	2	4	2	1	0	0	0	0	0	0	12
	407	0	2	2	0	4	4	1	0	1	1	1	0	0	0	16
	413	0	4	1	4	4	4	3	1	0	0	0	0	0	0	21
	876	0	1	0	1	3	4	0	1	0	0	0	0	0	1	11
																**mean score 15.4**
BuPV_ΔN^pro^_E1E2 CP7	406	0	0	0	1	3	2	0	0	0	0	0	0	0	0	6
live 1x	418	0	1	0	1	0	1	0	0	0	0	0	0	0	0	3
	893	0	0	0	0	4	0	0	0	0	0	0	0	0	0	4
	894	0	0	0	2	1	0	0	0	0	0	0	0	0	0	3
	898	0	0	0	0	0	0	0	0	0	0	0	0	0	0	0
																**mean score 3.2**
BuPV_ΔN^pro^_E1E2 CP7	405	0	0	0	0	1	2	0	0	0	0	0	0	0	0	3
inactivated 2x	410	0	0	0	0	0	0	1	0	0	0	0	0	0	0	1
	412	0	0	0	0	0	0	0	0	0	0	0	0	0	0	0
	414	0	0	0	0	0	0	0	0	0	0	0	0	0	0	0
	417	0	0	0	1	1	0	0	0	0	0	0	0	0	0	2
																**mean score 1.2**

## Data Availability

The data presented in this study are available in the article and the Supplementary Materials.

## Supplementary Materials

The following are available online at https://www.mdpi.com/article/10.3390/vaccines10010088/s1, Figure S1: In-vitro characterization of BuPV and BuPV_ΔNpro_E1E2 CP7. Single step growth kinetics in KOP-R cells at an m.o.i. of 1. Supernatants were harvested 0, 24, 48 and 72 h p.i. and titrated on Madin–Darby bovine kidney (MDBK) cells. Mean values of three representative experiments are displayed. Error bars represent standard deviations.
