# Supplementary figures and images for: Bungowannah Pestivirus Chimeras as Novel Double Marker Vaccine Strategy against Bovine Viral Diarrhea Virus
Source: Vaccines (Basel). 2022 Jan 7;10(1):88. doi: 10.3390/vaccines10010088 (PMC8778585; doi:10.3390/vaccines10010088)

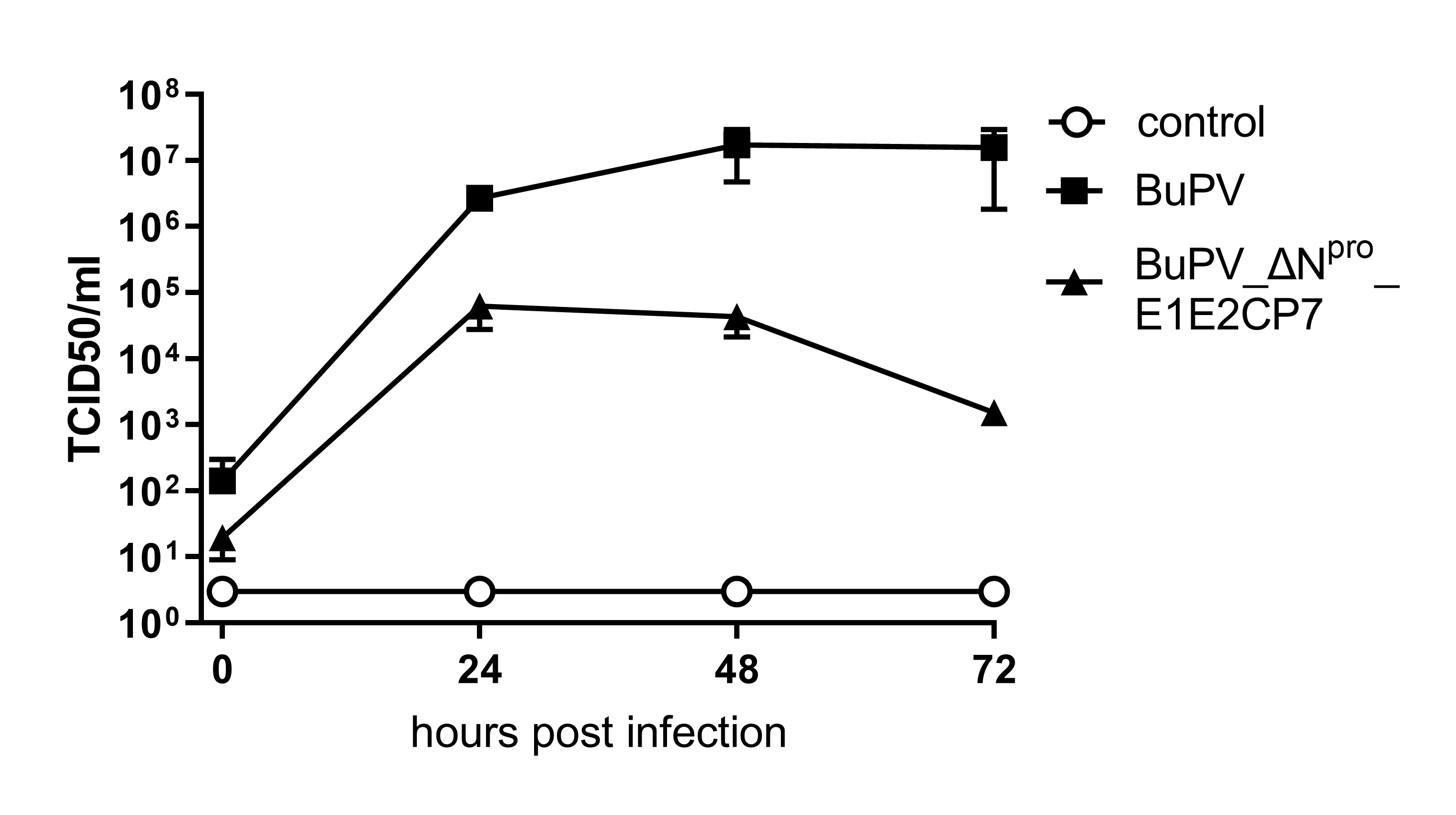

Supplement: Supplementary file 1 [file vaccines-10-00088-s001.zip › Fig S1.tif]
